# The Association between Survivorship Care Plans and Patient-Reported Satisfaction and Confidence with Follow-Up Cancer Care Provided by Primary Care Providers

**DOI:** 10.3390/curroncol29100577

**Published:** 2022-09-30

**Authors:** Alanna K. Chu, Brittany Mutsaers, Sophie Lebel

**Affiliations:** School of Psychology, University of Ottawa, Ontario, ON K1N 6N5, Canada

**Keywords:** cancer survivorship, survivorship care plans, follow-up care, cancer care, primary care, psychosocial oncology

## Abstract

Survivorship care plans aim to facilitate a smooth transition from tertiary to primary care settings after primary cancer treatment is completed. This study sought to identify the sociodemographic factors associated with receiving a survivorship care plan and examine the relationship between receiving a plan and confidence in follow-up care delivered by primary care providers. A cross-sectional analysis of the Canadian Partnership Against Cancer’s Experiences of Cancer Patients in Transition Study was conducted (n = 9970). Separate adjusted multinomial logistic regression models assessed the relationship between survivorship care plans and follow-up care outcomes. Proportion of survivors more likely to receive a survivorship care plan varied by numerous sociodemographic and medical factors, such as cancer type (colorectal and prostate), gender (male), and education (high school or less). In unadjusted and adjusted models, individuals who received a Survivorship Care Plan had significantly higher odds of: having felt their primary care providers were involved; agreeing that their primary care providers understood their needs, knew where to find supports and services, and were able to refer them directly to services; and were confident that their primary care provider could meet their follow-up care needs.

## 1. Introduction

Over the past decade, advances in life-prolonging treatments, such as immunotherapies, personalized medicine, and targeted therapies, have resulted in significant improvements in survival rates for many patients with cancer [1]. Survivorship care needs for cancer survivors are broad and complex, and range significantly depending on the clinical features of the cancer, the treatment, and the individual. Due to the cancer and its treatment, cancer survivors are at a higher odds for ongoing chronic medical conditions (e.g., fatigue, pain, cognitive dysfunction), and psychosocial and practical concerns (e.g., distress, anxiety, sexuality, roles in relationships, fear of cancer recurrence, transitioning back to work, etc.) [2,3].

Increased cancer survivorship has resulted in follow-up care demands that have surpassed the capacity for cancer centres. In response, the Institute of Medicine recommended that all patients should receive a survivorship care plan (SCP), sometimes called a follow-up care plan, upon discharge from primary cancer treatment to the community [4]. The aim of SCPs is to improve the quality, coordination, and continuity of care by providing adequate transitional information about cancer treatment and follow-up care from cancer specialist teams to primary care providers (PCPs) [4,5]. SCPs typically include a treatment summary, steps for follow-up care and management of side effects related to the cancer and its treatment, surveillance for recurrent disease, and health-promotion materials [3,6,7]. SCPs have been endorsed by numerous authorities in global cancer care (Pan-Canadian Guideline for Survivorship Services for Adult Cancer Populations; American Cancer Society; Centers for Disease Control), including Cancer Care Ontario (CCO) in Ontario, Canada. The SCP is a key component of CCO’s Follow-Up Model of Care (2019), which has the stated goals of providing survivors with high-quality care in appropriate settings, having providers to be engaged and supported in providing this care, and optimizing healthcare system resource utilization [5].

Since the conception of SCPs, there has been significant research examining a broad range of outcomes. The evidence for the efficacy of SCPs on long term health outcomes has often been found to be mixed and inconclusive [7,8]. For example, numerous descriptive studies and randomized clinical trials have shown little and inconsistent effect of SCPs on long-term patient reported health outcomes, such as psychological or cancer-specific distress, quality of life, and satisfaction with care [7]. A recent Cochrane Review found that the certainty of evidence for the efficacy of survivorship care plans was very low for outcomes such as health-related quality of life, anxiety, depression, or cost [8]. Other studies have found null or inconsistent results related to satisfaction with care, survivorship knowledge or functioning, continuity of care, cost effectiveness, and unmet needs [7,9,10,11,12]. These results have cause critics to question to the overall efficacy and utility of SCPs [7].

However, research on healthcare utilization and delivery has been more positive, with numerous studies finding beneficial effects of SCPs related to adherence to medical recommendations, increase PCPs self-reported knowledge, increased knowledge of PCPs role in follow-up care, and improvement in follow-up care satisfaction [7,13,14,15]. Additionally, many non-randomized studies have shown good feasibility related to generating and delivering SCPs, and acceptability amongst patients and healthcare workers [7]. Notably, numerous studies have found that patients who received SCPs were satisfied with the plan (e.g., found it useful, easy to understand), SCPs improved patients’ understanding of their follow-up care, and improved perceived coordination and knowledge of care [16,17]. Thus, it appears that studies should measure outcomes that are more proximally related to SCPs’ stated goals of improving the coordination of follow-up care, increasing understanding of provider roles, and increasing knowledge of follow-up care for patient and PCPs [7].

Beyond the range of outcomes measured, factors that may contribute to differences found across studies include heterogeneity of the administration and content of survivorship care plans across institutions or jurisdictions, variations in study designs, and differences in patient populations [6,7]. Existing studies typically look at one or two patient group(s) and/or at one institution or jurisdiction. Efficacy of SCPs at the population level are not yet well understood.

Additionally, little is known about the sociodemographic factors contributing to the probability of receiving an SCP or how these relate to the relationship between SCPs and patient reported satisfaction outcomes. This is important, as sociodemographic factors have been found to be related to follow-up care received by survivors. For example, lower socioeconomic status is associated with lower follow-up care discussions and care [18,19], Sociodemographic factors, such as ethnicity, age, income level, education level, and more have been identified as important factors related to disparities in health outcomes, as well as health-care access, utilization and satisfaction for patients with cancer and those with other chronic illnesses [18,20,21,22,23,24,25,26]. Thus, it is important to understand both whether sociodemographic and medical factors influence the odds of receiving an SCP in Canada, and whether the sociodemographic and medical factors affect the efficacy of SCP for survivors who receive them. It is also important to understand whether or not SCPs are significantly associated with follow-up care while controlling for sociodemographic and medical factors.

In order to address these gaps in the literature, this study will analyze an extensive pan-Canadian survey of recent cancer survivors. The goals of this study are to (1) examine the sociodemographic and medical factors related to receiving SCPs in Canada, and (2) compare self-reported outcomes related to involvement of PCPs in follow-up cancer care and confidence and satisfaction with follow-up care delivered by PCP in survivors who did or did not receive an SCP while controlling for sociodemographic and medical factors. It is hypothesized that sociodemographic factors and medical factors will affect the probability of receiving an SCP, and that SCPs will be associated with improved involvement, satisfaction and confidence with follow-up care provided by PCPs. Results will inform our current understanding of the real-world implementation and efficacy of SCPs in Canada and advise future areas of inquiry.

## 2. Methods

This study is a cross-sectional retrospective analysis of the Canadian Partnership Against Cancer’s (CPAC) Experiences of Cancer Patients in Transition Study conducted between June and October 2016. The Transitions Study was made possible by funding provided to the Canadian Partnership Against Cancer by Health Canada [27]. The survey focused on adolescent and young adult (AYA; ages 18–29) and adult (ages 30+) cancer survivors between 1 and 3 years following cancer treatment with a broad range of cancer diagnoses. Survey methodology is published by Fitch and colleagues (2019). The survey was designed using a conceptual framework of survivorship care needs based on a literature review [28] and consultations with cancer survivors, clinicians, and system leaders were conducted to guide the development of survey items. The survey aimed to answer the following three questions: (1) What are the needs of cancer survivors 1–3 years after treatment, (2) who are the most vulnerable cancer survivors, and (3) what factors/system resources are correlated with needs being met? Patients were selected from provincial registries based on eligibility criteria and medical records were linked by provincial cancer agency/registry staff to determine eligibility. Eligible participants were mailed a survey package from the provincial cancer agency/registry [27]. Data (n = 13,319) are openly available online on the Canadian Partnership Against Cancer website (https://www.systemperformance.ca/transitions-study/transition-study-questions/, accessed on 15 September 2020). Ethics approval was not required for secondary data analysis. The assumptions and/or calculations underlying the results were prepared by Alanna Chu and Sophie Lebel, University of Ottawa, and the responsibility for the use and interpretation of these data and their reporting is entirely that of the author(s).

### 2.1. Sociodemographic and Medical Variables

Sociodemographic and medical variables included in this study are as follows: metastatic disease status (yes/no), cancer type (blood/breast/colorectal/lymphoma/melanoma/prostate/other), age group (AYA/Adults), sex (male/female), rural or urban status, educational level (high school or less/undergraduate or college degree/graduate degree), income level (low ≤ CAD 25,000/medium = CAD 25,000–CAD 74,999/high ≥ CAD 75,000), marital status (single/married or partnered/separated or widowed), employment (unpaid work/employed/unemployed), immigration status (yes/no).

Immigration status (yes/no) is defined as being born outside of Canada (yes) or being born in Canada (no). In the survey, the options for sex included male (n = 6411), female (n = 6820), other (n = 7) and prefer not to answer (n = 81). The “other” category was removed as analyses were insufficiently powered. Employment status included unpaid work (i.e., retired, student, homemaker), employed (i.e., paid leave, paid sick leave, full-time employed, part-time employed), or unemployed. Those who selected multiple occupations were given attributed one status with the following priority: employed, unpaid, unemployed, no answer. Responses left blank, chose not to answer, and not applicable were removed from the analysis.

Cancer type was self-reported and cancer types with less than 400 respondents were included in the “other” category. Categories were as follows: lymphoma (Hodgkin’s, Diffused B-Cell lymphoma), blood (acute lymphocytic leukemia, myelogenous leukemia, non-Hodgkin’s lymphoma, other types of leukemia, other type of blood cancers), breast, colorectal, melanoma skin cancer, prostate cancer, and other cancers (bladder, brain/central nervous system, gynecological, sarcoma, stomach or esophagus, testicular, thyroid, bone, renal, kidney, liver, hepatic, lungs/respiratory, oral/tongue, throat, abdominal, ganglion, other). Participants who selected more than one cancer type (n = 477), selected “none” (n = 14), “I don’t know” (n = 5), or left response blank (n = 801) were excluded from the analysis.

### 2.2. Survivorship Care Outcome Variables

Outcome variables related to confidence and satisfaction with follow-up care included the following questions: (1) Since completing your cancer treatment, which physician has been in charge of overseeing your follow-up cancer care? (i.e., most responsible physician). Responses included (a) family doctor/general practitioner/nurse practitioner, (b) your oncologist, hematologist, surgeon, or other cancer specialist, (c) both. (2) How involved is your family doctor/general practitioner/nurse practitioner in your follow- up cancer care? Responses were measured on a four-point Likert scale ranging from very involved, somewhat involved, not very involved, and not at all involved. For the purposes of analysis, a binary variable was produced (involved/not involved). Participants who responded “no general practitioner” (GP), “not applicable” or chose not to answer were excluded from the analysis. (3) How much do you agree or disagree with the following statements about your family doctor/general practitioner/nurse practitioner when it comes to your follow-up cancer care? My family doctor/general practitioner/nurse practitioner… (a) …understands what I need when it comes to follow-up cancer care, (b) …knows where to find other supports and services to help in my follow-up cancer care, (c) …is able to refer me directly to other supports and services to help in my follow-up cancer care, and (d) I am confident that my family doctor/general practitioner/nurse practitioner can take care of my needs in follow-up cancer care. Responses were measured on a five-point Likert scale ranging from strongly agree, somewhat agree, neither agree nor disagree, somewhat disagree and strongly disagree. For the purposes of this categorical data analysis, a three-level variable was calculated (agree/neutral/disagree). Responses left blank, chose not to answer, and not applicable were removed from the analysis.

To address the first aim of the study (i.e., assess the relationship between receiving an SCP and sociodemographic and medical variables), bivariate analyses (i.e., chi-squared tests) were conducted for the relationship between receiving an SCP and each sociodemographic variable, and count, proportion and *p*-values from chi-squared tests were reported. Adjusted residuals for each chi-squared test were reported, and significance was calculated using a Bonferroni correction.

To address the second aim of the study (i.e., assess the relationship between receiving an SCP and the satisfaction and confidence with care outcomes), bivariate analyses (i.e., chi-squared tests) were conducted for the relationship between receiving an SCP and all satisfaction/confidence outcome variables listed above (i.e., Questions 1–3). Separate unadjusted multinomial logistic regressions [29] were conducted for the relationship between receiving an SCP and Questions (1) and (3) and a binomial logistic regression model was conducted for the relationship between receiving an SCP and Question (2). Analysis of Question (3) were conducted on a subset of participants who reported ‘involved’ or ‘not involved’ on Question (2), excluding participants who indicated that they did not have a GP, selected “not applicable” or chose not to answer, as to exclude presumably false ratings of PCPs for participants who do not have a PCP.

**Figure 1:**
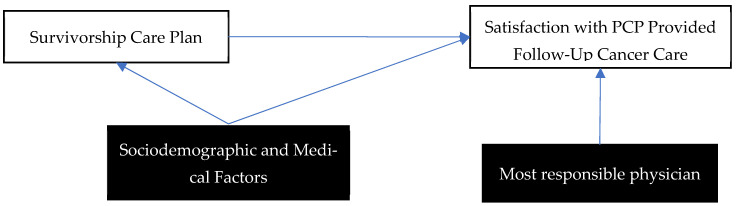
Directed Acyclic Graph on the Relationship between SCP and Satisfaction with PCP Delivered Follow-Up Cancer Care.

Finally, an adjusted multinomial logistic regression was completed controlling for all sociodemographic factors and medical variables. It is hypothesized that these factors may influence the odds of receiving an SCP and that these factors may also influence the involvement, confidence and satisfaction with follow-up care (Figure 1). Additionally, the most responsible physician variable was added to the adjusted multinomial logistic regressions as a covariate for Question (3), as it is hypothesized that an individual’s most responsible physician may influence the ratings of satisfaction with PCP delivered follow-up care (e.g., bias towards or against oncologists or PCPs based on familiarity with most responsible physician). Odds ratios were reported for all analyses. All data analysis was conducted using R statistical software.

## 3. Results

Bivariate analyses comparing sociodemographic factors and receiving an SCP vs. not receiving an SCP (Table 1) found significant differences for cancer type (higher proportion in colorectal and prostate cancer, and lower in blood, breast, lymphoma, melanoma and other cancers), age group (40.42% vs. 59.85% in AYA patients), marital status (42.65% vs. 57.35% in the single category), education level (38.91% vs. 61.09% graduate degree, 53.18% vs. 46.82% high school or less, and 44.23% vs. 55.77% undergraduate degree or college diploma), income level (44.27% vs. 55.73% high, 50.95% vs. 49.05% middle, and 48.38% vs. 51.62% low), and employment status (44.76% vs. 55.24% employed, 51.40% vs. 48.60% unpaid, and 43.43% vs. 56.57% unemployed). Survivors with breast cancer, lymphoma, and melanoma were less likely to receive an SCP, while survivors with colorectal and prostate cancer were more likely to report receiving an SCP. Participants who reported they were adults, married, had a high-school diploma or less, men, live in rural areas, middle income or unpaid were more likely to receive an SCP. Individuals who were AYA, single, female, had a graduate degree or undergraduate/college degree, lived in urban areas, or were employed were less likely to report receiving an SCP.

Bivariate analyses comparing receiving SCPs and outcome variables (Table 2) found significant differences between receiving an SCP and not receiving an SCP on all outcome variables.

Unadjusted models and multivariate models adjusting for all sociodemographic and medical variables (Table 3; Tables S1–S6) found significant relationships between SCPs and all outcome variables related to satisfaction and confidence with follow-up care provided by PCPs. Models found individuals who were given an SCP had significantly higher odds of having a general practitioner (GP; OR: 1.47, 95% CI: 1.34–1.61; aOR: 1.48, 95% CI: 1.32–1.67) or both an oncologist and GP (OR: 1.19, 95% CI: 1.08–1.32; aOR: 1.34, 95% CI: 1.18–1.53) responsible for follow-up cancer care compared to only a specialist/oncologist and having felt their PCP was involved (OR: 2.02, 95% CI: 1.85–2.20; aOR: 1.95, 95% CI: 1.75–2.17) compared to not involved. Additionally, those with an SCP had significantly higher odds of agreeing (compared to neutral) that their PCP understood their needs (OR: 2.45, 95% CI 2.17–2.76; aOR: 2.16, 95% CI: 2.13–3.01), knew where to find supports and services (OR: 1.90, 95% CI: 1.69–2.13; aOR: 1.74, 95% CI: 1.50–2.01), was able to refer them directly to supports and services (OR: 1.76, 95% CI: 1.56–1.99; aOR: 1.54, 95% CI: 1.32–1.78), and were confident that they could meet their follow-up care needs (OR: 2.03, 95% CI: 1.79–2.29; aOR: 1.83, 95% CI: 1.57–2.14).

## 4. Discussion

The results of this study provide preliminary evidence that sociodemographic and medical factors have a significant impact on the chances of receiving an SCP. Additionally, this study found that receiving an SCP is significantly associated with patient-reported involvement, satisfaction and confidence of follow-up care provided by PCPs. These results have important implications for how we understand and research the efficacy of SCPs in Canada.

This study found that likelihood of receiving an SCP was influenced by sociodemographic factors, including cancer type (increased for colorectal cancer and prostate cancer), age group, marital status, education, sex, rural/urban status, income level, education level, employment, and metastatic status. The relationship between SCPs and cancer type are expected, as cancers with traditionally high rates of incidence and survival (e.g., prostate cancer and colorectal cancer) tend to have higher rates of relevant programming (e.g., psychosocial supports, survivorship resources, advocacy, etc.) and thus may have a higher likelihood of receiving an SCP [7,30]. Sex-specific prevalence by cancer types (e.g., prostate cancer, breast cancer) may also account for the sex differences in receiving an SCP. AYA patients were less likely to receive an SCP compared to adults; this is important given that previous research has found that younger patients have higher survivorship needs and less satisfied with the transition [31].

Results found that respondents who were not sure about their metastatic status were less likely to have received an SCP, indicating possibly lower knowledge of cancer history. Survivors with high income and high education were less likely to receive SCP, and those with low income or unpaid employment were more likely to receive an SCP. These results are surprising given that patients with higher income and education levels, and who are employed, typically have lower barriers to access of cancer healthcare services [32,33], specifically access to PCP. Additionally, there was no association between immigration status and SCPs. These results warrant further inquiry to determine the underlying reasons for these differences, as this may indicate that SCPs are differentially delegated based on sociodemographic factors. Cultural context, diversity, and social determinates of health should be considered in the implementation of SCPs and in the investigation of the barriers and facilitators to accessing these types of intervention. Additionally, sociodemographic and medical factors should be considered in the ways in which we understand the various outcomes related to SCP in existing and future research.

Results from this study also provide preliminary evidence that SCPs are related to increased PCP involvement in follow-up cancer care and increased confidence and satisfaction with such care, even after controlling for sociodemographic and medical factors and most responsible physician. Respondents who received an SCP had significantly higher likelihood of having a PCP either partially or completely responsible for follow-up cancer care and had almost double the likelihood of reporting that their PCP was involved (compared to not involved) with their care. These results suggest that survivors who receive an SCP may be more likely to successfully transition from cancer centres to their PCP, which has important implications for cancer centres which are already overburdened and do not have the capacity to provide long term follow-up care for all cancer survivors.

Respondents who had received an SCP also had significantly higher likelihood of reporting that their PCPs understood their needs, knew and could refer them to relevant supports and services, and were confident in their ability to provide follow-up care. Additionally, participants whose most responsible physician were both oncologists and PCPs, or PCP alone were more likely to agree with questions related to confidence and satisfaction with PCP delivered follow-up care in the adjusted models, which may indicate a positive appraisal bias towards one’s more responsible physician. It is well documented that survivors who transition from cancer centres to primary care can lead to significant anxiety and fear about losing support and expertise of the cancer team [34]. These findings indicate that survivors feel confident and satisfied with the care they receive from their PCPs when they are their most responsible physician, which lends further evidence of PCPs ability to manage follow-up cancer care. Even after holding the effect of the most responsible physician constant, individuals who received SCPs were more likely to reported increased confidence and satisfaction with care. Although only a proxy for an individual’s experience, quality of life or behaviour change, the importance of satisfaction and confidence in care should not be underestimated as confidence in care is related to reduced distress and anxiety [35,36]. Thus, this provides key evidence towards the use and implementation of SCP in practice and is consistent with research examining the acceptability of survivorship care plans at the institutional level.

There are several limitations of the study. First, although this study utilized data from a large, Pan-Canadian study with diverse sociodemographic and economic backgrounds and histories, the study is not population representative and thus cannot be generalized to the larger Canadian survivor population. Given the scope of the study and the limitations of the data, we are unable to determine which factors in SCPs (e.g., treatment summary, surveillance procedures, placebo effects, etc.) and mode of delivery are related to the positive outcomes in satisfaction, involvement and confidence. SCPs are highly heterogenous across regions, hospitals, cancer types, and cancer programs, and thus these results provide preliminary evidence that SCPs broadly are related to positive outcomes. Future research should focus on the specific aspects of SCPs which increase confidence and satisfaction with PCP delivered follow-up care. SCPs are also often given to survivors as part of a package (e.g., education classes, discharge appointments) [37]. Thus, these results may also be driven by resources which often accompany SCPs, rather than the SCPs themselves. Lastly, this study provides preliminary evidence for the relationship between sociodemographic and medical factors, but these relationships should be explored and understood at granular level to determine the underlying drivers of these relationships.

Despite these limitations, the results of this study suggest that there may be significant benefits of SCPs for survivors transitioning from tertiary cancer centres back to primary care. Given that the provision of SCPs may depend on various sociodemographic and medical factors, further research should be enacted to understand for which patients SCPs are most beneficial and in what way. In a resource constrained environment, more should be performed to understand the social determinates of health and the individual follow-up care needs of cancer survivors and public health funding should be focused on delivering interventions as equitability and efficiently as possible.

## Figures and Tables

**Table 1:** Chi-squared analysis of sociodemographic factors and survivorship care plan (SCP).

	No SCP Received ^1^		SCP Received			
	n	Proportion	n	Proportion	Chi-Square Residuals	*p*-Value
N	5108		4862			
Cancer Type						<0.001
Blood	186	55.03	152	44.97	−1.33	
Breast	1700	55.27	1376	44.73	−5.15 *	
Colorectal	772	44.86	949	55.14	6.11 *	
Lymphoma	357	59.70	241	40.30	−4.16 *	
Melanoma	496	58.63	350	41.37	−4.36 *	
Prostate	904	43.86	1157	56.14	7.88 *	
Other	272	58.75	191	41.25	−3.21*	
Metastasis						0.022
No Mets	3902	50.67	3799	49.33	2.61	
Primary Mets	298	54.58	248	45.42	−1.57	
Secondary Mets	215	50.59	210	49.41	0.31	
Unsure	465	55.53	370	44.47	−2.55	
Age Groups						0.004
AYA ^2^	171	59.58	116	40.42	−2.88 *	
Adults	4919	50.96	4733	49.04	2.88 *	
Marital Status						<0.001
Divorced/Separated/Widowed	926	52.32	844	47.68	−0.99	
Married/Partnered	3703	50.37	3649	49.63	2.99 *	
Single	429	57.35	319	42.65	−3.48 *	
Education						<0.001
Graduate Degree	562	61.09	358	38.91	−6.28 *	
≤Highschool	2561	46.82	2909	53.18	9.89 *	
Undergraduate or College	1866	55.77	1480	44.23	−6.46 *	
Sex ^3^						<0.001
Female	2928	54.98	2398	45.02	−8.05 *	
Male	2156	46.88	2443	53.12	8.05 *	
Rural/Urban						0.003
Rural	1688	49.14	1747	50.86	3.02 *	
Urban	3346	52.33	3048	47.67	−3.02 *	
Income Level						<0.001
High	1483	55.73	1178	44.27	−5.11 *	
Middle	1962	49.05	2038	50.95	4.78 *	
Low	636	51.62	596	48.38	0.06	
Employment						<0.001
Employed ^4^	1934	55.24	1567	44.76	−5.86 *	
Unpaid ^5^	290	48.60	3076	51.40	6.48 *	
Unemployed	155	56.57	119	43.43	−1.80	
Immigration Status						0.825
Yes	824	50.99	792	49.01	−0.22	
No	4231	51.29	4018	48.71	0.22	

Note: all missing values excluded. ^1^ Respondents who selected that “not applicable” to having receiving an SCP (n = 2215) and missing values (n = 1134) excluded from analysis. ^2^ Adolescents and Youth Adults. ^3^ Other (n = 4) and prefer not to answer (n = 41) excluded due to low sample size. ^4^ Employed full-time, part-time, or on leave. ^5^ Homemaker, student or retired. * Statistical significance at the 0.05 level, adjusted using a Bonferroni correction.

**Table 2:** Bivariate Analysis of SCPs and Patient Reported Satisfaction Outcomes.

	No SCP Received ^1^		SCP Received		
	n	Proportion	n	Proportion	*p*-Value
Primary Care Provider					*p* < 0.001
General Practitioner	1442	44.80	1777	55.20	
Oncologist	1078	50.00	1078	50.00	
Both	2294	54.40	1923	45.60	
PCP involvement					*p* < 0.001
Not involved	1986	62.32	1201	37.68	
Involved	2840	45.05	3464	54.95	
No General Practitioner	135	58.95	94	41.05	
Understanding needs					*p* < 0.001
Disagree	584	71.05	238	28.95	
Neutral	955	66.32	485	33.68	
Agree	3001	44.49	3744	55.51	
Knows where to find supports and services					*p* < 0.001
Disagree	460	73.72	164	26.28	
Neutral	909	61.63	566	38.37	
Agree	2974	45.63	3543	54.37	
Able to refer me directly to supports and services					*p* < 0.001
Disagree	439	73.53	158	26.47	
Neutral	832	60.51	543	39.49	
Agree	3059	46.33	3543	53.67	
Confidence in ability to provide follow-up care					*p* < 0.001
Disagree	871	70.41	366	29.59	
Neutral	814	62.23	494	37.77	
Agree	2915	44.54	3629	55.46	

Note: all missing values excluded. ^1^ Respondents who selected that “not applicable” to having receiving an SCP (n = 2215) and missing values (n = 1134) excluded from analysis.

**Table 3:** Multinomial and Binomial Logistic Regression Analysis for SCPs and Patient Reported Satisfaction Outcomes.

	Received an SCP					
	Unadjusted Model			Adjusted Model		
	Risk Ratio	95% CIs	*p*-Value	Risk Ratio	95% CIs	*p*-Value
**Clinician responsible for follow-up care**						
Oncologist	(ref.)			(ref.)		
Both	1.47	(1.34, 1.61)	*p* < 0.001	1.49	(1.33, 1.68)	*p* < 0.001
General Practitioner	1.19	(1.08, 1.32)	*p* < 0.001	1.35	(1.18, 1.54)	*p* = 0.001
**PCP Involvement**						
Not involved	(ref)			(ref)		
Involved	2.02	(1.85, 2.20)	*p* < 0.001	1.96	(1.76, 2.18)	*p* < 0.001
**PCP understands needs**						
Neutral	(ref)			(ref)		
Disagree	0.78	(0.64, 0.94)	*p* = 0.009	1.03	(0.82, 1.30)	*p* = 0.844
Agree	2.45	(2.17, 2.76)	*p* < 0.001	2.17	(1.87, 2.53)	*p* < 0.001
**PCP knows where to find supports and services**						
Neutral	(ref.)			(ref.)		
Disagree	0.55	(0.44, 0.68)	*p* < 0.001	0.71	(0.55, 0.92)	*p* = 0.009
Agree	1.90	(1.69, 2.13)	*p* < 0.001	1.74	(1.50, 2.01)	*p* < 0.001
**PCP is able to refer me directly to supports and services**						
Neutral	(ref.)			(ref.)		
Disagree	0.53	(0.43, 0.65)	*p* < 0.001	0.65	(0.50, 0.85)	*p* = 0.001
Agree	1.76	(1.56, 1.99)	*p* < 0.001	1.53	(1.32, 1.78)	*p* < 0.001
**Confidence ability for PCP to provide follow-up care**						
Neutral	(ref.)			(ref.)		
Disagree	0.67	(0.57, 0.80)	*p* < 0.001	0.78	(0.63, 0.95)	*p* = 0.016
Agree	2.03	(1.79, 2.29)	*p* < 0.001	1.83	(1.56, 2.14)	*p* < 0.001

Note: Each adjusted analysis controls for sociodemographic factors including: metastatic disease status (yes/no), cancer category (blood/breast/colorectal/lymphoma/melanoma/prostate/other cancer), age group (Adolescent and Young Adult/Adults), sex (male/female), rural or urban status, educational level (high school or less/undergraduate or college degree/graduate degree), income level (low/medium/high), marital status (single/married or partnered/separated or widowed), employment (unpaid work, employed, unemployed), immigration status (yes/no). Adjusted and non-adjusted analysis used a subset of the sample that excluded individuals who identified as “not having a general practitioner” or not. All missing values excluded. See Tables S1–S6 for full adjusted models.

## Data Availability

This analysis is based on the Canadian Partnership Against Cancer’s Experiences of Cancer Patients in Transition Study survey. The Transitions Study was made possible by funding provided to the Canadian Partnership Against Cancer by Health Canada. The assumptions and/or calculations underlying the results were prepared by Alanna Chu and Dr. Sophie Lebel at the University of Ottawa and the responsibility for the use and interpretation of these data and their reporting is entirely that of the author(s). Data is openly available at: https://www.systemperformance.ca/transition-study/,accessed on 15 September 2020.

## Supplementary Materials

The following supporting information can be downloaded at: https://www.mdpi.com/article/10.3390/curroncol29100577/s1, Table S1: Multinomial Logistic Regression for Clinician Type; Table S2: Binomial Log Regression of Perceptions of Involvement with Follow-Up Cancer Care; Table S3: GP Understands what I need when it comes to follow-up cancer care; Table S4: GP knows where to find other supports and services to help in my follow-up cancer care; Table S5: General practitioner is able to refer me directly to other supports and services to help in my follow-up cancer care; Table S6: Confidence in GP to meet follow-up care needs.
