# Supplementary material for: The Association between Survivorship Care Plans and Patient-Reported Satisfaction and Confidence with Follow-Up Cancer Care Provided by Primary Care Providers
Source: Curr Oncol. 2022 Sep 30;29(10):7343–54. doi: 10.3390/curroncol29100577 (PMC9600139; doi:10.3390/curroncol29100577)
Supplement: Supplementary file 1 [file curroncol-29-00577-s001.zip › curroncol-1864313-supplementary.pdf]

Table S1. Multinomial Logistic Regression for Clinician Type

	Both				General Practitioner			
	RR	95%CI		P-Value	RR	95% CI		P-Value
(Intercept)	0.53	0.36	0.77	0.001	0.08	0.04	0.16	0.000
No Careplan Received	(ref)				(ref)			
Careplan Received	1.48	1.32	1.67	0.000	1.34	1.18	1.53	0.000
Blood Cancer	(ref)				(ref)			
Breast Cancer	2.48	1.82	3.38	0.000	12.93	7.20	23.20	0.000
Colorectal Cancer	2.04	1.51	2.77	0.000	7.78	4.34	13.96	0.000
Lymphoma	1.09	0.77	1.54	0.622	1.44	0.73	2.84	0.298
Melanoma	1.20	0.86	1.69	0.282	6.31	3.46	11.52	0.000
Other Cancer	1.33	0.90	1.95	0.151	3.95	2.02	7.73	0.000
Prostate	1.78	1.31	2.43	0.000	4.36	2.41	7.90	0.000
No Mets	(ref)				(ref)			
Primary Mets	1.13	0.88	1.44	0.335	0.79	0.59	1.07	0.133
Secondary Mets	1.08	0.83	1.42	0.564	0.62	0.42	0.91	0.015
Unsure Mets	1.20	0.97	1.50	0.099	1.32	1.03	1.70	0.029
Adults	(ref)				(ref)			
AYA	0.67	0.46	0.98	0.040	0.49	0.28	0.85	0.011
Female	(ref)				(ref)			
Male	0.74	0.62	0.87	0.000	0.74	0.60	0.91	0.004
Rural	(ref)				(ref)			
Urban	0.76	0.67	0.86	0.000	0.78	0.68	0.89	0.000
Undergraduate or College	(ref)				(ref)			
Graduate Degree	1.01	0.84	1.23	0.880	0.73	0.57	0.93	0.011
Highschool or Less	1.09	0.96	1.23	0.195	1.17	1.01	1.35	0.034
Middle Income	(ref)				(ref)			
High Income	0.84	0.73	0.96	0.012	0.90	0.77	1.05	0.188
Low income	1.06	0.89	1.27	0.519	0.89	0.72	1.10	0.296
Single	(ref)				(ref)			
Divorced/ Separated/ Widowed	0.86	0.67	1.11	0.261	1.09	0.81	1.48	0.563
Married/ Partner	0.97	0.78	1.23	0.828	1.08	0.81	1.43	0.600
Employed								
Unpaid Work	0.89	0.79	1.01	0.076	0.95	0.82	1.09	0.471
Unemployed	0.70	0.50	1.00	0.047	0.80	0.53	1.20	0.276

Analytical sample n = 6659

Table S2. Binomial Log Regression of Perceptions of Involvement with Follow-Up Cancer Care

		Involved		
	**RR**	**95% CI**		**P-Value**
(Intercept)	1.15	0.80	1.65	0.464
No Careplan Received	(ref)			
Careplan Received	1.95	1.75	2.17	0.000
Blood Cancer	(ref)			
Breast Cancer	1.93	1.45	2.57	0.000
Colorectal Cancer	1.65	1.24	2.19	0.001
Lymphoma	1.54	1.11	2.13	0.009
Melanoma	0.93	0.69	1.26	0.640
Other Cancer	1.72	1.20	2.47	0.003
Prostate	1.49	1.12	1.98	0.007
No Mets	(ref)			
Primary Mets	1.04	0.83	1.31	0.753
Secondary Mets	0.94	0.72	1.22	0.622
Unsure Mets	1.05	0.86	1.29	0.614
Adults	(ref)			
AYA	0.57	0.40	0.80	0.001
Female	(ref)			
Male	0.89	0.76	1.04	0.129
Rural	(ref)			
Urban	0.81	0.72	0.91	0.000
Undergraduate or College	(ref)			
Graduate Degree	0.90	0.76	1.07	0.235
Highschool or Less	1.14	1.01	1.28	0.033
Middle Income	(ref)			
High Income	0.84	0.74	0.95	0.005
Low income	1.33	1.12	1.60	0.002
Single	(ref)			
Divorced/ Separated/ Widowed	0.91	0.71	1.16	0.448
Married/ Partner	0.97	0.78	1.21	0.776
Employed	(ref)			
Unpaid Work	0.97	0.86	1.08	0.543
Unemployed	0.70	0.51	0.95	0.023

Analytic sample n = 6725

Table S3. GP Understands what I need when it comes to follow-up cancer care

	Disagree				Agree			
	RR	95%CI		P-Value	RR	95% CI		P-Value
(Intercept)	1.47	0.73	2.94	0.279	2.40	1.44	3.99	0.001
No SCP Received	(ref)				(ref)			
Received SCP	1.02	0.81	1.29	0.844	2.16	1.85	2.51	0.000
Oncologist	(ref)				(ref)			
Oncologist and PCP	0.77	0.58	1.01	0.057	2.53	2.13	3.01	0.000
PCP	1.11	0.83	1.48	0.501	2.22	1.82	2.72	0.000
Blood Cancer	(ref)				(ref)			
Breast Cancer	0.99	0.58	1.68	0.966	1.42	0.96	2.09	0.077
Colorectal Cancer	0.84	0.49	1.43	0.519	1.26	0.86	1.84	0.238
Lymphoma	0.91	0.50	1.65	0.759	1.26	0.82	1.94	0.284
Melanoma	0.87	0.48	1.56	0.636	1.54	1.02	2.34	0.042
Other Cancer	0.87	0.44	1.70	0.682	1.42	0.87	2.31	0.161
Prostate	0.67	0.38	1.17	0.157	1.25	0.86	1.83	0.245
No Mets	(ref)				(ref)			
Primary Mets	1.14	0.75	1.75	0.533	0.84	0.62	1.14	0.263
Secondary Mets	1.46	0.86	2.47	0.161	0.99	0.69	1.42	0.949
Unsure Mets	1.07	0.71	1.59	0.756	0.86	0.65	1.13	0.273
Adults	(ref)				(ref)			
AYA	1.25	0.67	2.34	0.483	0.85	0.52	1.39	0.521
Female	(ref)				(ref)			
Male	0.62	0.45	0.85	0.003	1.02	0.82	1.27	0.869
Rural	(ref)				(ref)			
Urban	0.91	0.71	1.15	0.410	0.85	0.73	1.00	0.044
Undergraduate or College	(ref)				(ref)			
Graduate Degree	1.18	0.83	1.67	0.368	1.14	0.89	1.46	0.315
Highschool or Less	0.84	0.66	1.07	0.165	0.95	0.81	1.12	0.568
Middle Income	(ref)				(ref)			
High Income	0.95	0.74	1.22	0.702	0.90	0.76	1.07	0.251
Low income	0.77	0.52	1.15	0.199	1.16	0.91	1.48	0.233
Single	(ref)				(ref)			
Divorced/ Separated/ Widowed	0.71	0.43	1.18	0.191	0.72	0.50	1.02	0.066
Married/ Partner	0.80	0.51	1.25	0.322	0.77	0.56	1.06	0.114
Employed	(ref)				(ref)			
Unpaid Work	0.64	0.51	0.81	0.000	1.02	0.87	1.19	0.838
Unemployed	1.80	0.96	3.39	0.068	1.61	0.97	2.68	0.065

Analytic sample n = 6260

Table S4. GP knows where to find other supports and services to help in my follow-up cancer care

	Disagree				Agree			
	RR	95%CI		P-Value	RR	95% CI		P-Value
(Intercept)	0.85	0.40	1.80	0.675	1.78	1.08	2.91	0.022
No SCP Received	(ref)				(ref)			
Received SCP	0.71	0.55	0.92	0.009	1.74	1.50	2.01	0.000
Oncologist	(ref)				(ref)			
Oncologist and PCP	0.73	0.54	0.98	0.038	2.08	1.76	2.46	0.000
PCP	1.02	0.74	1.39	0.920	1.82	1.50	2.20	0.000
Blood Cancer	(ref)				(ref)			
Breast Cancer	0.83	0.47	1.47	0.532	1.33	0.91	1.94	0.147
Colorectal Cancer	0.95	0.53	1.68	0.852	1.30	0.90	1.90	0.167
Lymphoma	0.99	0.52	1.90	0.987	1.37	0.89	2.10	0.152
Melanoma	0.78	0.41	1.48	0.445	1.53	1.02	2.32	0.042
Other Cancer	0.87	0.41	1.83	0.717	1.37	0.84	2.24	0.207
Prostate	0.90	0.49	1.65	0.725	1.47	1.01	2.15	0.045
No Mets	(ref)				(ref)			
Primary Mets	1.37	0.87	2.15	0.174	0.92	0.68	1.24	0.571
Secondary Mets	1.22	0.65	2.28	0.534	1.10	0.76	1.59	0.608
Unsure Mets	1.26	0.83	1.92	0.273	0.91	0.70	1.20	0.518
Adults	(ref)				(ref)			
AYA	1.72	0.82	3.58	0.148	1.71	0.99	2.94	0.054
Female	(ref)				(ref)			
Male	0.47	0.33	0.67	0.000	0.90	0.73	1.12	0.361
Rural	(ref)				(ref)			
Urban	1.01	0.78	1.31	0.946	0.82	0.70	0.95	0.011
Undergraduate or College	(ref)				(ref)			
Graduate Degree	0.80	0.54	1.20	0.278	1.00	0.79	1.27	0.997
Highschool or Less	0.90	0.70	1.17	0.440	0.97	0.82	1.13	0.665
Middle Income	(ref)				(ref)			
High Income	1.16	0.88	1.52	0.290	0.98	0.83	1.16	0.804
Low income	1.07	0.70	1.62	0.763	1.35	1.05	1.72	0.018
Single	(ref)				(ref)			
Divorced/ Separated/ Widowed	0.82	0.48	1.39	0.452	0.88	0.63	1.22	0.432
Married/ Partner	1.00	0.62	1.60	0.998	1.08	0.80	1.46	0.625
Employed	(ref)				(ref)			
Unpaid Work	0.75	0.58	0.96	0.025	1.25	1.07	1.46	0.004
Unemployed	2.07	1.11	3.86	0.022	1.48	0.92	2.39	0.107

Analytic sample n = 6011

Table S5. General practitioner is able to refer me directly to other supports and services to help in my follow-up cancer care

	Disagree				Agree			
	RR	95%CI		P-Value	RR	95% CI		P-Value
(Intercept)	0.91	0.43	1.90	0.797	1.38	0.84	2.26	0.203
No SCP Received	(ref)				(ref)			
Received SCP	0.65	0.50	0.85	0.001	1.54	1.32	1.78	0.000
Oncologist	(ref)				(ref)			
Oncologist and PCP	0.78	0.57	1.05	0.102	2.24	1.88	2.66	0.000
PCP	0.97	0.70	1.33	0.829	1.85	1.52	2.26	0.000
Blood Cancer	(ref)				(ref)			
Breast Cancer	0.87	0.50	1.51	0.621	1.62	1.11	2.37	0.012
Colorectal Cancer	0.94	0.54	1.67	0.843	1.52	1.05	2.21	0.027
Lymphoma	0.93	0.49	1.74	0.811	1.39	0.91	2.12	0.126
Melanoma	0.87	0.45	1.65	0.666	2.06	1.35	3.13	0.001
Other Cancer	0.51	0.24	1.12	0.094	1.37	0.85	2.22	0.194
Prostate	0.89	0.49	1.63	0.710	1.58	1.08	2.30	0.018
No Mets	(ref)				(ref)			
Primary Mets	1.31	0.83	2.07	0.252	0.81	0.60	1.10	0.177
Secondary Mets	1.27	0.69	2.34	0.435	0.94	0.65	1.35	0.734
Unsure Mets	1.69	1.12	2.56	0.013	0.98	0.74	1.30	0.883
Adults	(ref)				(ref)			
AYA	1.82	0.88	3.74	0.104	1.56	0.93	2.61	0.091
Female	(ref)				(ref)			
Male	0.49	0.34	0.70	0.000	1.08	0.86	1.34	0.507
Rural	(ref)				(ref)			
Urban	1.11	0.85	1.45	0.450	0.86	0.73	1.01	0.063
Undergraduate or College	(ref)				(ref)			
Graduate Degree	0.77	0.50	1.18	0.232	1.14	0.89	1.47	0.298
Highschool or Less	0.91	0.70	1.18	0.478	1.04	0.88	1.22	0.636
Middle Income	(ref)				(ref)			
High Income	1.02	0.77	1.35	0.876	0.93	0.79	1.11	0.439
Low income	0.94	0.61	1.44	0.764	1.31	1.02	1.69	0.033
Single	(ref)				(ref)			
Divorced/ Separated/ Widowed	0.75	0.44	1.27	0.279	0.97	0.70	1.36	0.879
Married/ Partner	0.90	0.56	1.43	0.644	1.15	0.85	1.56	0.358
Employed	(ref)				(ref)			
Unpaid Work	0.83	0.64	1.07	0.149	1.27	1.08	1.49	0.003
Unemployed	1.00	0.52	1.89	0.989	0.98	0.64	1.49	0.913

Analytic sample n = 5961

Table S6. Confidence in GP to meet follow-up care needs

	Disagree				Agree			
	**RR**	**95%CI**		**P-Value**	**RR**	**95% CI**		**P-Value**
(Intercept)	1.44	0.76	2.74	0.266	1.91	1.13	3.22	0.016
No SCP Received	(ref)				(ref)			
Received SCP	0.78	0.63	0.95	0.016	1.83	1.57	2.14	0.000
Oncologist	(ref)				(ref)			
Oncologist and PCP	0.69	0.54	0.88	0.002	2.46	2.06	2.94	0.000
PCP	1.09	0.82	1.45	0.557	3.15	2.52	3.93	0.000
Blood Cancer	(ref)				(ref)			
Breast Cancer	0.79	0.48	1.29	0.348	1.08	0.71	1.64	0.732
Colorectal Cancer	0.66	0.40	1.09	0.107	1.02	0.68	1.55	0.908
Lymphoma	0.92	0.53	1.58	0.755	1.03	0.65	1.64	0.890
Melanoma	0.62	0.36	1.05	0.078	1.02	0.65	1.58	0.937
Other Cancer	0.86	0.46	1.60	0.625	1.26	0.74	2.12	0.396
Prostate	0.72	0.44	1.19	0.204	1.11	0.73	1.68	0.625
No Mets	(ref)				(ref)			
Primary Mets	1.31	0.89	1.91	0.168	0.78	0.57	1.08	0.134
Secondary Mets	1.33	0.81	2.16	0.256	1.02	0.69	1.50	0.913
Unsure Mets	1.44	1.01	2.06	0.043	0.91	0.68	1.22	0.537
Adults	(ref)				(ref)			
AYA	1.20	0.66	2.18	0.541	1.01	0.61	1.67	0.981
Female	(ref)				(ref)			
Male	0.67	0.50	0.90	0.007	1.02	0.81	1.28	0.847
Rural	(ref)				(ref)			
Urban	1.00	0.81	1.24	0.981	0.87	0.74	1.03	0.101
Undergraduate or College	(ref)				(ref)			
Graduate Degree	1.17	0.86	1.59	0.321	0.96	0.74	1.23	0.739
Highschool or Less	0.90	0.72	1.12	0.346	1.06	0.90	1.26	0.490
Middle Income	(ref)				(ref)			
High Income	0.88	0.70	1.10	0.259	0.93	0.78	1.11	0.410
Low income	0.91	0.64	1.28	0.571	1.30	1.01	1.69	0.046
Single	(ref)				(ref)			
Divorced/ Separated/ Widowed	1.30	0.83	2.03	0.254	0.97	0.69	1.37	0.877
Married/ Partner	1.54	1.03	2.31	0.035	1.19	0.88	1.61	0.269
Employed	(ref)				(ref)			
Unpaid Work	0.77	0.62	0.95	0.015	1.07	0.91	1.27	0.400
Unemployed	1.85	1.01	3.40	0.046	1.62	0.95	2.77	0.076

Analytic sample n = 6316
